# Supplementary material for: The impact of oxytocin on emotion recognition and trust: Does disordered eating moderate these relationships?
Source: PLoS One. 2024 May 31;19(5):e0303824. doi: 10.1371/journal.pone.0303824 (PMC11142561; doi:10.1371/journal.pone.0303824)
Supplement: Attachment — Submitted filename: Oxytocin_Response_letter_PlosOne_FINAL.docx [file pone.0303824.s001.docx]

PONE-D-23-32813

The Impact of Oxytocin on Emotion Recognition and Trust: Does Disordered Eating Moderate these Relationships?

PLOS ONE

Dear Prof. Hidenori Yamasue

Thank you for providing us with yours and the reviewers’ suggestions as to how we can improve our manuscript entitled: PONE-D-23-32813 *" The Impact of Oxytocin on Emotion Recognition and Trust: Does Disordered Eating Moderate these Relationships?“*

We have carefully reviewed the comments and have revised the manuscript accordingly. In the manuscript, we have highlighted with track changes all the changes we have undertaken. Our responses to the reviewers’ concerns are given in a point-by-point manner below.

**Reviewer #1**

Minor comments
**Comment 1:** Page 4, line 81: it would be helpful to have more demographics from these studies (gender, age,..) to identify possible influencing factors.

**Response 1**: This information has now been deleted because one of reviewer 2 requested/recommended to shorten the introduction.

**Comment 2:** Page 8, line 189: "ed", probably a typo in the sentence.

**Response 2:** Thanks for spotting this. We have fixed the typo in text.

**Comment 3:** Page 8, line 190: I lack some background knowledge on oxytocin/ED and body satisfaction in the introduction.

**Response 3:** We’ve added a few lines addressing this under ‘the Current study’. The new information added now reads as:*” Specifically, given the co-occurrence of increased body dissatisfaction and state anxiety [7] in females with high levels of disordered eating and that oxytocin has been shown to decrease state anxiety [8], the current study theorised that if state anxiety decreased following an oxytocin intervention state body satisfaction would increase.”*

**Comment 4:** Page 8, line 196: Now it seems like you expect them to have ‘better performance at a trust game’, but you would expect ‘increased trust’, not better game performance, right?

**Response 4:** Thank you for pointing this out. We have now rephrased this sentence. It now reads as: *“We therefore hypothesised that compared to the placebo condition, (Hypothesis 1) participants in the oxytocin condition would score higher on emotion recognition and increased trust, and (Hypothesis 2) participants in the oxytocin condition would report decreased state anxiety and negative affect and increased state body satisfaction at post-intervention assessment.”*

**Comment 5:** Page 8, 199-204: this is basically repeating the sentence before, seems redundant.

**Response 5:** Thank you – we edited this section for conciseness.

**Comment 6:** Page 9, line 228: Did oral contraceptives affect the results? What about non-oral contraceptives, menstrual cycle (or menopause)?

**Response 6:** Thank you for pointing out these two important influencing factors. Women who take oral contraceptives may sustain elevated oxytocin levels over prolonged periods. This heightened presence of oxytocin could diminish the impact of the hormone's typically moderate releases, potentially altering its usual physiological effects.

Garforth, B., Degnbol, H., Terris, E.T. *et al.* Elevated plasma oxytocin levels and higher satisfaction with life in young oral contraceptive users. *Sci Rep* **10**, 8208 (2020). <https://doi.org/10.1038/s41598-020-64528-w>

Similarly, oxytocin plays a crucial role in both psychological and physiological processes, which often display distinct patterns based on sex, suggesting an interaction between oxytocin and sex hormones. Additionally, the levels of oxytocin may vary throughout the menstrual cycle, in tandem with shifts in the concentrations of female sex hormones.

Engel S, Klusmann H, Ditzen B, Knaevelsrud C, Schumacher S. Menstrual cycle-related fluctuations in oxytocin concentrations: A systematic review and meta-analysis. Front Neuroendocrinol. 2019 Jan; 52:144-155. doi: 10.1016/j.yfrne.2018.11.002. Epub 2018 Nov 17. PMID: 30458185.

Surprisingly only 22% of the current sample used the oral contraceptive pill and only 29% of the participants reported that they were in days 3 to 12 of their menstrual cycle.

We have provided more details about these variables that were reported in table 1 now in the text on Page 17. The new information now reads as*:” Table 1 shows that the two groups were generally equivalent at baseline; chi-square tests confirmed no significant differences between the oxytocin and placebo groups, for the demographic (ethnicity, main language spoken at home, paid employment, education, marital status, and clinical variables (use of the contraceptive pill, other medication, self-reported psychiatric diagnoses, current and lifetime ED, day 3 to12 of menstrual cycle). However, participants in the oxytocin group scored significantly higher on eating pathology than the control group (32% vs 15%). Independent samples t-tests revealed no significant differences between groups for age and BMI. Fourteen percent of the sample (N = 21) had a high EAT-26 total score, and 10% of the sample (N = 15) reported having a history of an ED, with similar ratios across the two groups. There were only 3 participants with a lifetime self-reported psychiatric condition other than an ED (2 with mixed depression/anxiety and 1 with anxiety). Only 22% of the overall sample were currently using the contraceptive pill and 29% were currently on day 3 to 12 of their menstrual cycle.”*

We have now outlined these influencing factors and the fact that we only assessed oral contraceptives as two additional limitations in the discussion. This section on Page 26 now reads as:” *Finally, our sample were young females of whom 22% took oral contraceptives. It should be noted that women who take oral contraceptives may sustain elevated oxytocin levels over prolonged periods. This heightened presence of oxytocin could have diminished the impact of the moderate releases of oxytocin in our oxytocin group, potentially altering its usual physiological effects in this group [68]. Furthermore, we only asked about oral contraceptive usage and therefore do not know what percentage of our sample used other non-oral contraceptives. Similarly, it should be acknowledged that oxytocin may vary throughout the menstrual cycle, in tandem with shifts in the concentrations of female sex hormones and these fluctuations could have impacted our findings [69].*

We don’t believe that menopause is an issue in the current sample since the mean age of our sample was 18.79.

**Comment 7:** Page 11, line 271: Elaborate on why the 'intranasal spray' section was interleaved in the task and questionnaire sections.

**Response 7:** The participants taking part in this experiment received research credits in exchange for taking part in the current study. This is why all the assessment had to be done in one session. Trait-based and sociodemographic questions were strategically placed at the beginning of the assessment, prior to the intervention, under the premise that these variables are independent and should not vary because of the intervention. We have outlined this on Page 15 (please refer to page numbers in the manuscript version with tracked changes)*:” Next, participants completed the immediately post-intervention measures, involving the demographic questionnaire and the trait measures EAT-26 (considered independent from the effects of the intervention).”*

**Comment 8:** Page 13, line 304: What was the scientific argumentation of not including baseline measures of these primary outcome measures?

**Response 8:** The scientific argumentation of not including baseline measures of these primary outcome measures was that for these tasks practice effects can be found and also including a baseline assessment would have made it very difficult to disentangle the practice effects from the intervention effects. We have made this clearer on this page by adding the following sentence: “*The TASIT-R and trust game were administered exclusively post-intervention, rather than at baseline, to prevent potential practice effects on these tasks from confounding the actual effects of the oxytocin or saline nasal sprays.”*

**Comment 9:** Page 15, 358: So, all participating investigators did have access to the unblinding?

**Response 9:** No, only the principal investigator (first author) had access to the unblinding in case a participant a negative reaction to the intervention. The authors who were involved with data collection (O.M and S.F) did not have access to the unblinding. We have made this clearer on Page 15 by adding the following text*:” The investigators (O.M and S.F) involved in data collection were blinded.”* These student investigators were also not blinded when they conducted the analyses. This information was /isoutlined in the manuscript.

**Comment 10:** Page 16, 399: Was this significantly different, seeing it is more than double? Reading further, I noticed that the EAT scores indeed significantly differed between the intervention groups (p=0.02). This should be investigated further and highlighted more in the manuscript.

**Response 10:** We have clarified in text that this is significantly different for eating pathology. This information about significance levels is also contained in Table 1. While EAT scores might ordinarily be considered as a potential covariate or moderator to add for further analyses (to assess impact of this baseline group difference), we do not do so in the current study since EAT scores are already planned for use in analyses (Hypothesis 3). Given the lack of moderating effect of EAT scores, it appears that the group difference may not have had much effect in the current study. It is also reassuring that there were no other significant differences observed (see Table 1 and preliminary analysis subsection).

**Comment 11:** Page 17, line 407: ‘Preliminary analyses’: are these not the final analyses?

**Response 11:** We had used the terms ‘Preliminary Analyses’ and ‘Main Analyses’ to distinguish analyses for context (former) and those that directly address research hypotheses (latter). To make this clearer we have tried to make these titles more informative by adding the following titles:” 1.) *Preliminary Analyses: Tests of differences pre-intervention and 2.)* *Main analyses: Hypotheses testing.*

**Comment 12:** Page 17, line 416: More informative subtitles would be appreciated.

**Response 12:** We’ve edited the subtitle to highlight the key results.

This new subtitle is now worded as*:” Hypothesis 1 - Oxytocin and Better Performance on the Recognition of Positive Emotions”* We have also rephrased the subtitle for Hypothesis 3 testing. It now reads as*:” Hypothesis 3 – Moderation Effects of Eating Pathology”.*

**Comment 13:** Page 17, line 420: ‘Post-manipulation’: do the authors mean post-intervention?

**Response 13:** We’ve changed the wording to post-intervention for consistency.

**Comment 14:** Page 18, line 449: surprised and neutral, are these considered as positive emotions?

**Response 14:** Yes, according to the TASIT-R, which is a well validated measure they are.
**Comment 15:** Page 19, line 468-470: I lack some references here.

**Response 15:** We’ve added relevant references to support the statement. “*Our finding is also consistent with research involving healthy individuals [28], which reported that a single dose of oxytocin administration improved emotion recognition performance in both male and female participants [9, 29].”*
**Comment 16:** Page 22, 525-526: “the current study theorised that if state anxiety decreased following an oxytocin intervention state body satisfaction would increase.” This was not found though, so this hypothesis might be more relevant to mention in the methods or even in the introduction.

**Response 16:** We’ve moved statements relating to this to the introduction section.

**Reviewer #2:**

**Comment 18:** The current Introduction and Discussion are too long. Especially, the Introduction should be shortened to clearly state the interesting, novelty and relevance of current study aim.

**Response 18:** We’ve made edits to shorten these sections and to highlight the focus of the study.

**Comment 19:** How to exclude candidates with psychiatric conditions. Please clarify the definition of psychiatric condition and the methodology to diagnose them.

**Response 19:** This information was already included in the participant section on Page 9*:” Exclusion criteria* ***(self-reported)*** *included: a.) males; b.) aged <18 or >30; c.) being pregnant/trying to become pregnant; d.) breast feeding/breast-fed in the last two months; e.) heavy smokers (> 5 cigarettes per day) and/or heavy drinkers (>14 standard drinks per week); f.) having* ***a current substance use disorder or other psychiatric condition other than an ED*** *and g.) a history of heart disease, cardiac disorder, neurological disorder, or allergic reaction to preservatives (food additives).”* We have slightly rephrased this section so that it is now hopefully clear that the other psychiatric condition refers to any other psychiatric condition than an ED.

None of the participants presented with a current psychiatric diagnosis and only 3 with a lifetime diagnosis (two with depression and anxiety and one with only anxiety).

We have added this information to the participant section on Page 17-18*:” There were only 3 participants with a lifetime self-reported psychiatric condition other than an ED (2 with mixed depression/anxiety and 1 with anxiety).”*

**Comment 20:** The experiments for current study and data collection were conducted from Oct 2016 to Dec 2017. It is noted the long delay to submit the paper since the last data collection. When was the data analysed?

**Response 20:** The lag in publishing this data was because the primary author had been on parental leave 3 times since the data had been collected; once in 2017, once in 2019 and once in 2022. Taking care of three small children under the age of 6 in addition to working full time in a teaching and research position and having clinical responsibilities, unfortunately did not allow for a faster turn around and write of this manuscript. The data was analysed in 2017/2018 by two Master students, and they used these analyses to write up their Master thesis. The data was afterwards re-analysed by a seasoned statistician for the purpose of this publication.

**Comment 21:** In page 11, it was described that the placebo was normal saline. It should be likely to differentiate the placebo and oxytocin for the participants because of their different smells. Please specify and report the degree to keep blindness among the participants.

**Response 21:** Neither the Oxytocin nor the Saline intervention had a smell and therefore it is extremely unlikely that the non-existing smell could have influenced the findings. We have added this information to this section by including the following information*:” The intranasal sprays, which were both odorless, were contained in identical bottles with labels stating the participant code (i.e., 1, 2, 3 etc.). This number was linked to the condition (i.e., oxytocin or placebo intervention).”*

**Comment 22:** Please consider employing appropriate correction for multiple comparisons in the statistical analyses.

**Response 22:** We have elaborated on our approach to multiple comparisons in the Data Analysis plan section. The following information was added on Page 17: *“Although a range of outcome measures was tested, we retained alpha criterion at .05 rather than correcting for multiple comparisons. Given the paucity of studies exploring the effects of oxytocin in this context, there is a risk that correction for Type I error rate increases risk of Type II errors [56-58), prematurely terminating a viable line of inquiry.”*

[56] Feise, R.J. (2002). Do multiple outcome measures require p-value adjustment? *BMC Medical Research Methodology, 2*, 8. Doi: 10.1186/1471-2288-2-8

[57] Rothman, K.J. (1990). No adjustments are needed for multiple comparisons. Epidemiology, 1(1), 43-46.

[58] Rothman, K.J. (2010). Curbing type 1 and type 2 errors. European Journal of Epidemiology, 25(4), 223-224. Doi: 10.1007/s10654-010-9437-5

We have also added this as another potential limitation in the discussion on Page 26:” *Fourth, while the observed effect sizes and statistically significant results for the emotion recognition and state positive emotion findings are promising, it is important to approach these findings with caution. The possibility of Type I errors, arising from multiple comparisons, necessitates replication of these results in further studies to confirm their validity.”*

**Comment 23:** How to estimate the required sample size. Please clarify the methodology to decide the target sample size.

**Response 23:** We apologise for leaving this out of the initial submission. Our initial power was based on comparable calculations from a previous study with the identical experimental condition, aiming for a target sample of 150 (75 per arm). This information has been added to the method's Data Analysis component. This new section on Page 17 now reads as*:” Sample size targets for the present study were based on assumption of a moderate between-group difference for experimental and control conditions (consistent with calculations of 9) for our outcome measures. Setting alpha at .05 (two-tailed), power = .80, and Cohen’s d > .45, we set a target of 75 per arm (150 total sample).*

**Comment 24:** The results for the analyses of modulating effect of ED symptoms on efficacy of oxytocin should be reported in more details.

**Response 24:** We have included a table with the salient statistics for the interaction effects reported (Table 3) rather than including for all parameters. This enables concise presentation of information relevant for hypothesis 3.

We are grateful to the Editor and reviewers for their time and suggestions which have greatly improved the manuscript. We hope the revised version is now suitable for publication in PloSONE and look forward to hearing from you in due course.

Yours sincerely on behalf of the authors
