# Supplementary material for: The impact of oxytocin on emotion recognition and trust: Does disordered eating moderate these relationships?
Source: PLoS One. 2024 May 31;19(5):e0303824. doi: 10.1371/journal.pone.0303824 (PMC11142561; doi:10.1371/journal.pone.0303824)
Supplement: Attachment — Submitted filename: Oxytocin_Response_letter_PlosOne_29042024IK.docx [file pone.0303824.s002.docx]

PONE-D-23-32813 R1

The Impact of Oxytocin on Emotion Recognition and Trust: Does Disordered Eating Moderate these Relationships?

PLOS ONE

Dear Prof. Hidenori Yamasue

Thank you for providing us with yours and the reviewers’ suggestions as to how we can improve our manuscript entitled: PONE-D-23-32813R1 *" The Impact of Oxytocin on Emotion Recognition and Trust: Does Disordered Eating Moderate these Relationships?“*

We have carefully reviewed the comments and have revised the manuscript accordingly. In the manuscript, we have highlighted with track changes all the changes we have undertaken. Our responses to the reviewers’ concerns are given in a point-by-point manner below.

**Reviewer #1:**

**Comment 1:** I believe 'preliminary' might not be the most suitable term to use.

**Response 1:** We have changed the wording for these analyses. The subheading now specifies these analyses as*:” Differences in outcome variables between the oxytocin and control group at pre-intervention.”* We have also deleted the “Main Analyses” and now only refer to *“Hypotheses Testing”.*

**Comment 2:** Page 18, line 449: surprised and neutral, are these considered as positive
emotions?
I'm still concerned about the classification of "surprised" and "neutral" emotions as positive in the TASIT-R, as this seems to oversimplify the complex nature of these emotions (not-negative does not mean positive). Emotions like surprise can vary greatly depending on context—being pleasant when associated with good news, but negative if linked to shock or fear. Similarly, neutrality might be seen positively as calmness or negatively as indifference. The TASIT-R, while well-validated for assessing social perception, might not fully capture these nuances, especially given that emotional reactions can be highly subjective and context-dependent. Some additional nuance would be appropriate here.

**Response 2:** We appreciate the feedback from the reviewer and agree that these emotions can have a range of different connotations. Unfortunately, we can’t change the scoring of the TASIT-R. We have however, outlined this as a further limitation in the discussion section. This new limitation on Page 24 now reads as*:” Fourth, it is important to acknowledge that the perception of emotions, such as surprise, can exhibit considerable variability contingent upon the circumstances. For instance, surprise may evoke positive sentiments when linked with delightful news, whereas it may evoke negative reactions when associated with shock or fear. Similarly, neutrality can be construed positively, reflecting a sense of calmness, or negatively, suggesting indifference. Despite the comprehensive validation of the TASIT-R for assessing social perception, it may not fully capture these intricate nuances, primarily due to the subjective and context-dependent nature of emotional responses.”*

**Reviewer #2:**

**Comment 3:** My previous comment on the initial submission mean that the authors should clarify how to confirm that none of the participants presented with a current psychiatric diagnosis. Did you utilize diagnostic tools such as SCID? Who conducted the assessment of psychiatric diagnosis? Psychiatrist? Psychologist? Or just self-report? Please clarify and report them.

**Response 3:** These diagnoses were based on self-report. We had already included this previously on Page 7 (self-reported) has now been highlighted to clarify this: *” Exclusion criteria (****self-reported****) included: a.) males; b.) aged <18 or >30; c.) being pregnant/trying to become pregnant; d.) breast feeding/breast-fed in the last two months; e.) heavy smokers (> 5 cigarettes per day) and/or heavy drinkers (>14 standard drinks per week); f.) having a current substance use disorder or any other psychiatric condition other than an ED and g.) a history of heart disease, cardiac disorder, neurological disorder, or allergic reaction to preservatives (food additives). Exclusionary criteria were selected based on safety reasons (i.e., oxytocin can induce contractions in pregnancy) and on findings that these factors can interfere with the effectiveness of oxytocin [48].”*

**Comment 4:** It should be helpful to report the time period for analyses.

**Response 4:** The time periods for the analyses have now been added to page 14. *“Initial analyses were first conducted in 2017/2018 for two independent Master theses (O.M and S.F.) and later re-run for the current article in 2022 by a seasoned statistician (M.F).”*

**Comment 5:** It was known that Syntocinon have some smells. Therefore, most of previous studies have employed placebo containing all inactive ingredient to avoid any subjective effects of the substances other than those caused by oxytocin. Utilizing saline as placebo should be considered as a limitation.

**Response 5:** Thank you for this further suggestion. We have now added this as a further limitation in the discussion. On Page 24/25 we have added the following information: *“Seventh, the choice of saline as a placebo in this study may introduce a further limitation, as it may not completely replicate the inert properties of a placebo containing all inactive ingredients. Future studies should therefore employ placebos containing all inactive ingredients to mitigate potential subjective effects of substances other than oxytocin.”*

We are grateful to the Editor and reviewers for their time and suggestions which have greatly improved the manuscript. We hope the revised version is now suitable for publication in PloSONE and look forward to hearing from you in due course.

Yours sincerely on behalf of the authors
